# Identification of type III effectors modulating the symbiotic properties of *Bradyrhizobium vignae* strain ORS3257 with various *Vigna* species

**DOI:** 10.1038/s41598-021-84205-w

**Published:** 2021-03-01

**Authors:** Pongpan Songwattana, Clémence Chaintreuil, Jenjira Wongdee, Albin Teulet, Mamadou Mbaye, Pongdet Piromyou, Djamel Gully, Joel Fardoux, Alexandre Mahougnon Aurel Zoumman, Alicia Camuel, Panlada Tittabutr, Neung Teaumroong, Eric Giraud

**Affiliations:** 1 School of Biotechnology, Institute of Agricultural Technology, Suranaree University of Technology, Nakhon Ratchasima, 30000 Thailand; 2 IRD, Laboratoire des Symbioses Tropicales et Méditerranéennes, UMR 113, IRD/CIRAD/INRAE/Université de Montpellier/SupAgro, Campus de Baillarguet, TA-A82/J, 34398 Montpellier Cedex 5, France; 3 IRD, Laboratoire Commun de Microbiologie, UR040, ISRA, UCAD, Centre de Recherche de Bel Air, Dakar, Senegal; 4 Département de Biologie Végétale, University Cheikh Anta Diop, Dakar, Senegal

**Keywords:** Microbiology, Plant sciences

## Abstract

The *Bradyrhizobium vignae* strain ORS3257 is an elite strain recommended for cowpea inoculation in Senegal. This strain was recently shown to establish symbioses on some *Aeschynomene* species using a cocktail of Type III effectors (T3Es) secreted by the T3SS machinery. In this study, using a collection of mutants in different T3Es genes, we sought to identify the effectors that modulate the symbiotic properties of ORS3257 in three *Vigna* species (*V. unguiculata*, *V. radiata* and *V. mungo*). While the T3SS had a positive impact on the symbiotic efficiency of the strain in *V. unguiculata* and *V. mungo*, it blocked symbiosis with *V. radiata*. The combination of effectors promoting nodulation in *V. unguiculata* and *V. mungo* differed, in both cases, NopT and NopAB were involved, suggesting they are key determinants for nodulation, and to a lesser extent, NopM1 and NopP1, which are additionally required for optimal symbiosis with *V. mungo*. In contrast, only one effector, NopP2, was identified as the cause of the incompatibility between ORS3257 and *V. radiata*. The identification of key effectors which promote symbiotic efficiency or render the interaction incompatible is important for the development of inoculation strategies to improve the growth of *Vigna* species cultivated in Africa and Asia.

## Introduction

Thanks to the ability of legume plants to interact symbiotically with nitrogen fixing rhizobia, they play a major agronomical and ecological role. This interaction results in the formation of symbiotic organs, called nodules, generally on the root system, in which the bacteria fix nitrogen for the plant’s benefit and, in exchange, receive carbon sources.

The establishment of a successful symbiosis relies on a complex signaling dialogue between the two partners thereby enabling their mutual recognition, the triggering of the programmes that lead to the formation of the nodule and its infection, and the weakening of the plant immune system allowing the plant to tolerate a large intracellular bacterial population inside the nodule cells^1^.

The Nod factors (NF) synthetized by the rhizobia in response to the perception of plant flavonoid signal, are recognised as the first “key” signal governing nodulation and infection processes^2^. Beyond the NF signals, other bacterial components are important for the successful establishment of the symbiosis, including exopolysaccharides (EPS), lipopolysaccharides (LPS), capsular polysaccharides (KPS), and cyclic β-glucans^3,4^. These surface polysaccharides can act directly as a symbiotic signal or may be modified to avoid the induction of plant triggered immunity (PTI) which normally occurs when the plant recognises a PAMP signal^5,6^.

More surprisingly, to cope with the plant immune system, similar to plant and animal pathogens, rhizobia use T3SS to interact with their host^7^. The T3SS is a complex needle-like machine that allows injection of specialised bacterial effector proteins directly into the host cell^8^. These effectors modulate key host cell functions, most of which are related to the suppression of host immunity but others can modulate plant hormone signaling, metabolism or organelle function^9^.

Not all rhizobia possess a T3SS, but surprisingly, this secretory machinery is widespread among rhizobia belonging to the *Bradyrhizobium* genus^10^. Indeed, a phylogenomic study, based on a large number of bradyrhizobial genome sequences currently available showed that more than 90% of the nodulating bradyrhizobia strains contained not only *nod* genes but also a T3SS^10^. In fact, it appears that the *nod* and T3SS genes share the same evolutionary history, bradyrhizobia acquired the two gene clusters simultaneously via horizontal transfer of a symbiotic island containing both. The systematic conservation of the T3SS in almost all nodulating *Bradyrhizobium* strains, suggests that, like *nod* genes, T3SS genes play an important role in the symbiotic process.

The rhizobial type III effector proteins (T3Es), including those called Nop for “Nodulation outer proteins”, can play a positive, negative or neutral role in the symbiotic process depending on the host plant and the rhizobium^11,12^. They can promote bacterial infection and nodulation by repressing specific defense responses of plants activated by the recognition of bacterial signals (PAMP), or conversely, they can make the interaction incompatible if they are recognized by immune receptors of the plants [resistance proteins (R)] leading to activation of effector-triggered immunity (ETI)^13,14^.

More recently, it has been shown that some legume species of the *Aeschynomene* genus and also the cultivar *Glycine max* cv. Enrie are nodulated by *Bradyrhizobium* strains even if NF synthesis is disrupted^15,16^. In this case, the establishment of the interaction requires that the bacteria has a functional T3SS, evidence that certain T3Es can also directly activate nodulation in some legumes by bypassing the NF signal.

To better understand which effectors are important for the establishment of this NF-independent, T3SS-dependent symbiosis, we almost exhaustively mutated the predicted effector gene repertoire (23 out of the 27 putative T3Es) in *Bradyrhizobium* sp. strain ORS3257, which is one of the most efficient strains in triggering the NF-independent symbiotic process in *A. indica*^17^. Analysis of the symbiotic properties of the mutants demonstrated that in this strain, T3SS-dependent symbiosis relies on a cocktail of at least five effectors which play synergistic and complementary roles in nodule organogenesis, and in the infection and repression of plant immune responses. Among them, we identified the nuclear-targeted ErnA effector, which is highly conserved among bradyrhizobia, as a key actor of nodule organogenesis^17^.

Interestingly, the strain ORS3257, which was originally isolated from a root nodule of *Vigna unguiculata*, is one of the two dominant genetic types of *Bradyrhizobium* strains nodulating cowpea at different sites in Senegal^18,19^. Considering that this genome has 98.07% average nucleotide identity (ANI) with that of *B. vignae* LMG28791 type strain^20^ (calculated using http://enve-omics.ce.gatech.edu/ani/), this strain belongs to the *B. vignae* species. Furthermore, the strain was shown to be one of the most promising inoculants because it induces efficient nitrogen fixing nodules on various *V. unguiculata* cultivars and it is a good competitor for nodule occupancy^18,21^. However, nothing is known about the importance of *nod,* T3SS and T3Es genes in the symbiotic properties of this strain with its natural host plant.

In this study, we took advantage of a collection of mutants of ORS3257 strain to identify which genes are relevant for the symbiotic efficiency of this strain with *V. unguiculata*. Furthermore, because the strain has the potential to be used as inoculant, we also examined the symbiotic properties of the mutants on two other *Vigna* species, *V. radiata* (mung bean) and *V. mungo* (urad bean), which are cultivated in Asia.

## Results

### The T3SS of *B. vignae* ORS3257 strain has a different impact on symbiosis depending on the *Vigna* species considered

**Figure 1:**
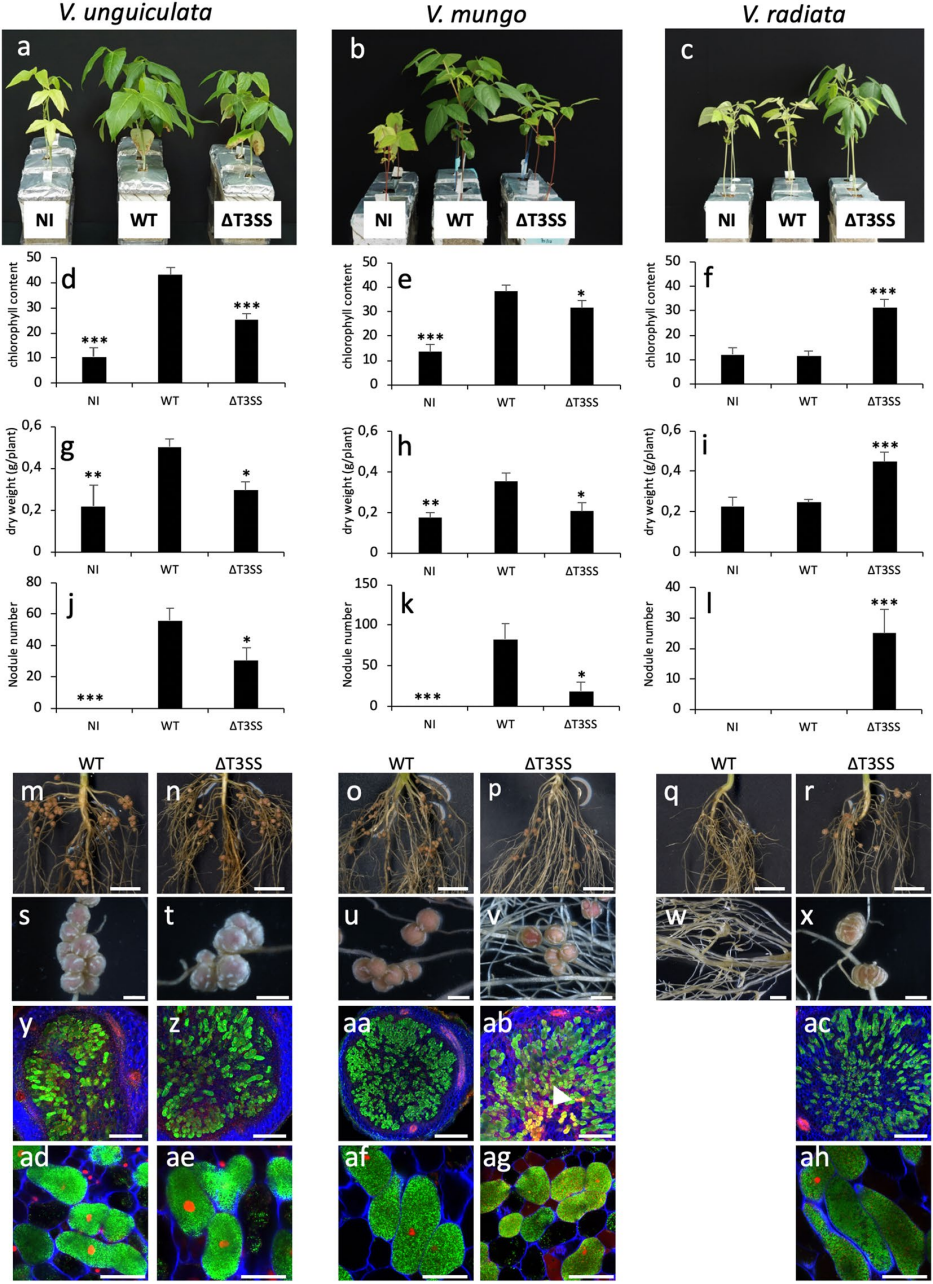
Symbiotic properties of *Bradyrhizobium vignae* ORS3257 strain (WT) and its derivative T3SS mutant (∆T3SS) in different *Vigna* species. (**a**–**c**) Comparison of the growth of the plants (aerial part), non-inoculated (NI) or inoculated with ORS3257 wild-type strain (WT) or its derivative T3SS mutant (∆*T3SS*) at 21 days after inoculation; (**d**–**f**) Leaf chlorophyll content; (**g**–**i**) Plant dry weight; (**j**–**l**) Number of nodules per plant. The experiment was carried out in duplicate with five plants per condition. *P < 0.05, **P < 0.01, and ***P < 0.001, significant differences between WT ORS3257 and the T3SS mutant using a nonparametric Kruskal–Wallis test. (**m**–**x**) View of the root and the nodules induced by ORS3257 strain and its derivative T3SS mutant (∆*T3SS*). (Scale bars, m to r, 1 cm; s to x, 2 mm). (**y**–**ah**) Cytological analysis of the nodules induced by strain WT ORS3257 and ∆T*3SS* observed by confocal microscopy after staining with SYTO 9 (green: live bacteria), calcofluor (blue: plant cell wall), and propidium iodide (red; infected plant nuclei and dead bacteria or bacteria with compromised membranes). (Scale bars, y to ac, 200 µm; ad to ah, 50 µm). White arrowhead in AB shows the necrotic zone.

In the first approach, we used three *Vigna* species to compare the symbiotic properties of the ORS3257 WT strain and its T3SS derivative mutant (3257∆*T3SS*) obtained by deletion of the main *rhc* gene cluster (from *nopB* to *rhcU*) (Supplementary Fig. S1). Interestingly, in the three plant species tested, we observed a strong effect of the T3SS deletion but this effect differed with the species. As shown in Fig. 1a,b,d,e,g,h,j,k, the *V. unguiculata* and *V. mungo* plants inoculated with the 3257∆*T3SS* mutant had lower chlorophyll content, lower growth, as well as fewer nodules than plants inoculated with the WT strain. These observations show that T3SS plays a positive role in the symbiotic efficiency of the strain in these two plant species.

In contrast, while the WT strain could not induce nodules on the *V. radiata* species, 3257∆*T3SS* formed nodules that were functional in terms of benefits for plant growth and leaf chlorophyll content (Fig. 1c,f,i,l).

To go deeper into the characterisation of the effect of the T3SS mutation, we also performed cytological analysis of the nodules elicited by the mutant. As shown in Fig. 1m–x, the nodules elicited by the 3257∆*T3SS* mutant displayed no apparent disorders whatever the species tested, i.e. the nodules were the characteristic pink color of leghemoglobin, evidence for their ability to fix nitrogen. Furthermore, confocal observations showed that the nodule cells of the central tissue were perfectly infected intracellularly by viable bacteria as revealed by the green colour of the Cyto9 stain (Fig. 1y–ah). The only notable observation was the presence of yellow autofluorescence zones in some rare nodules of *V. mungo,* suggesting the accumulation of phenolic compounds due to plant defence reactions (Fig. 1ab).

Taken together these data indicate that the T3SS contributes to the symbiotic efficiency of *B. vignae* on *V. mungo* and *V. unguiculata* while it renders incompatible the symbiotic interaction with *V. radiata* most probably by plant recognition of a specific T3E inducing ETI.

Considering that the T3SS has been shown to bypass the requirement of NF signalling during the interactions of some *Bradyrhizobium* strains including ORS3257 with some tropical legumes^15,16^, we constructed a mutant in which the *nodABC* genes were deleted and analysed its symbiotic properties. The ability of the 3257∆*nodABC* mutant to form nodules on *V. mungo* and *V. unguiculata* was found to be completely aborted. Like the WT strain, it did not also induce nodules on *V. radiata*. These data indicate that the symbiotic process between ORS3257 and *Vigna* species (at least for *V. mungo* and *V. unguiculata*) depends on NFs.

### Effectors NopT and NopAB play a major positive role in the interaction between ORS3257 and *V. mungo* and *V. unguiculata*

**Figure 2:**
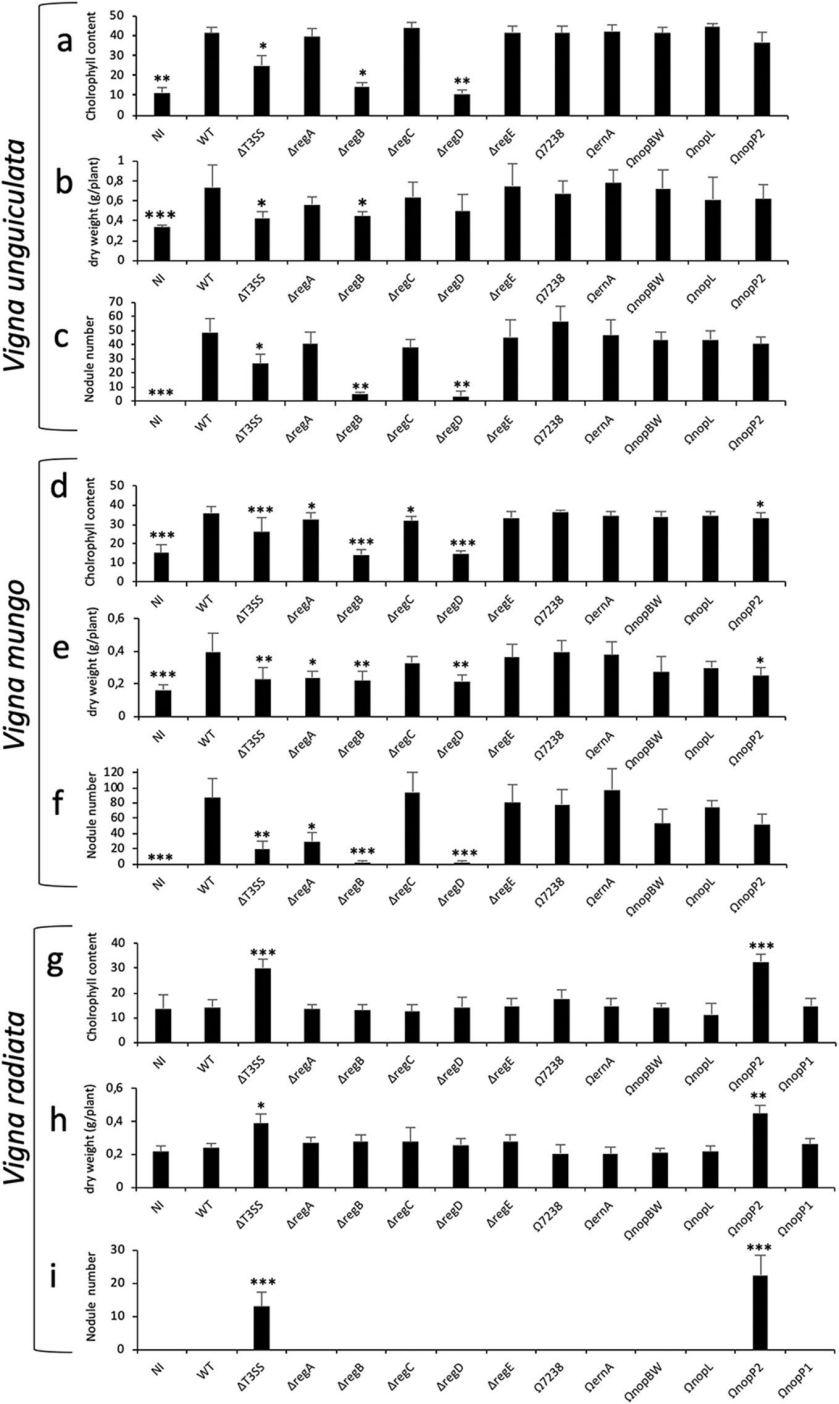
Identification of T3Es in *Bradyrhizobium vignae* ORS3257 strain that modulate the symbiotic interaction with *V. unguiculata, V. mungo* and *V. radiata.* (**a**,**d**,**g**) Leaf chlorophyll content of the plants inoculated with ORS3257 wild-type strain (WT) and its mutant derivatives at 21 days after inoculation; (**b**,**e**,**h**) Plant dry weight; (**c**,**f**,**i**) Number of nodules per plant. The experiment was carried out in duplicate with five plants per condition. *P < 0.05, **P < 0.01, and ***P < 0.001, significant differences between WT ORS3257 and the mutants using a nonparametric Kruskal–Wallis test.

To identify which T3Es have a positive impact on the symbiotic relationship between ORS3257 and *V. unguiculata* and *V. mungo*, we analysed the symbiotic properties of the T3Es ORS3257 mutants we had previously constructed^17^. This consisted of a collection of ten mutants, five deletion mutants in which genomic regions containing several clustered T3E genes were deleted by double crossing over, and five insertional mutants in specific T3E genes (*nopBW*, *nopL*, *nopP2*, *ernA*, and *Brad7238*) obtained by insertion of the non-replicative pVO155 vector by single crossing over (Supplementary Fig. S1). These ten mutants include 23 out of the 27 effectors predicted in silico in the ORS3257 genome^17^. Out of the 10 tested mutants, two mutants (3257∆*regB* and 3257∆*regD*) were drastically impacted in the two *Vigna* species, while two additional mutants (3257∆*regA*, and 3257Ω*nopP2*) displayed a weak phenotype only in *V. mungo* (Fig. 2a–f).

Given the symbiotic defects observed in the 3257∆*regB* and 3257∆*regD* mutants that were even greater than those observed in the ∆*T3SS* mutant, we analysed sub-mutants of these two regions to identify the effector(s) responsible for the phenotype.

**Figure 3:**
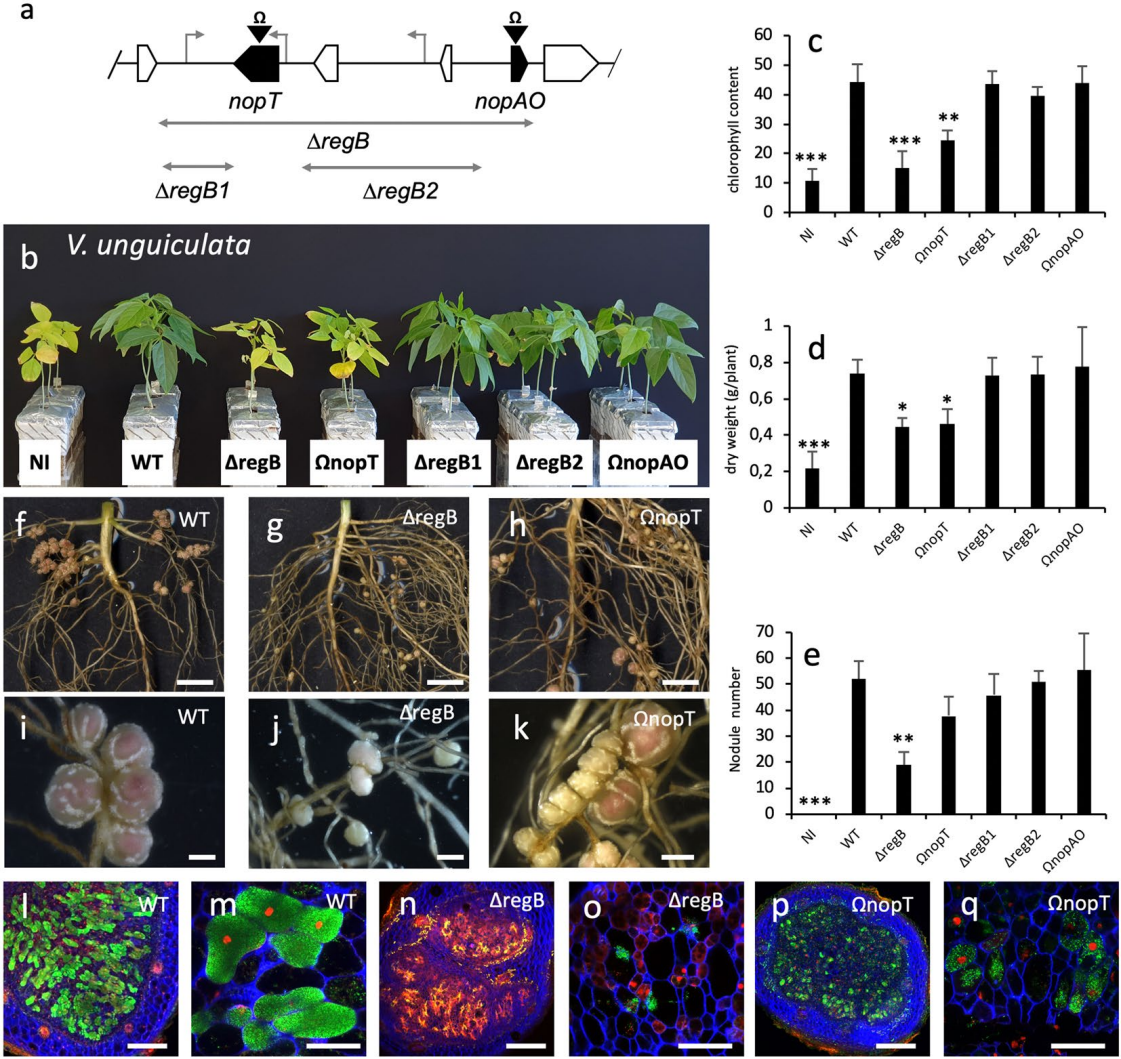
Symbiotic properties on *V. unguiculata* of the ORS3257 mutants in the different effectors and the regions encompassing the *tts* boxes identified in region B. (**a**) Genetic organisation of the putative effectors and *tts* boxes identified in region B. The deleted regions in the mutants are indicated by double direction arrows. The insertion mutants are indicated by black arrowheads carrying the Ω sign. In black, putative effector genes; gray arrows, *tts* boxes. (**b**) Comparison of the growth of the plants (aerial part), non-inoculated (NI) or inoculated with ORS3257 wild-type strain (WT) or its derivative mutants at 21 days after inoculation. (**c**) Leaf chlorophyll content. (**d**) Plant dry weight; (**e**) Number of nodules per plant. The experiment was carried out in duplicate with five plants per condition. *P < 0.05, **P < 0.01, and ***P < 0.001, significant differences between WT ORS3257 and its derivative mutants using a nonparametric Kruskal–Wallis test. (**f**–**k**) View of the root and the nodules induced by strain ORS3257 and its derivative mutants. (Scale bars: f to h, 1 cm; i to k, 2 mm). (**l**–**q**) Cytological analysis of the nodules induced by strain ORS3257 and its derivative mutants observed by confocal microscopy after staining with SYTO 9 (green: live bacteria), calcofluor (blue: plant cell wall), and propidium iodide (red: infected plant nuclei and dead bacteria or bacteria with compromised membranes). (Scale bars, l, n, p, 200 µm; m, o, q, 50 µm).

Region B contains two putative effector genes (*nopT* and *nopAO*) as well as two additional *tts* boxes with no clearly defined downstream coding sequence (Fig. 3a). Analysis of four available sub-mutants of this region corresponding to two insertional mutants in *nopT* and *nopAO* (Ω*nopT* and Ω*nopAO*) and two deletion mutations (∆*regB1* and ∆*regB2*) encompassing the region surrounding the two *tts* boxes showed that only the Ω*nopT* mutant had a symbiotic defect in the two *Vigna* species (Fig. 3b–e and Supplementary Fig. S2b–e). Nevertheless, it revealed that the phenotype of the Ω*nopT* mutant was less drastic than the one of the ∆*regB* mutant. In particular, while the ∆*regB* mutant induced only a few white nodules in the two *Vigna* species, the Ω*nopT* mutant formed more nodules and these nodules consisted of a mixture of white and pink nodules in *V. unguiculata* and mainly pink nodules in *V. mungo* (Fig. 3f–k and Supplementary Fig. S2f–k). Furthermore, cytological analysis revealed that the ∆*regB* nodules were completely necrotic and/or very poorly infected, with a notable problem of releasing of bacteria into the host cells, i.e. the bacteria were not uniformly dispersed in the cell but remained clustered together (Fig. 3l–o and Supplementary Fig. S2l–o). While the white Ω*nopT* nodules on *V. unguiculata* or the pink Ω*nopT* nodules on *V. mungo* were generally better infected, some plant cells were filled with uniformly dispersed viable bacteria (Fig. 3p,q and Supplementary Fig. S2p,q). These data suggest that the absence of *nopT* in the ∆*regB* mutant was largely responsible for the observed ∆*regB* phenotype but the absence of another as yet unidentified genetic determinant in this region likely also contributes to the observed defects.

**Figure 4:**
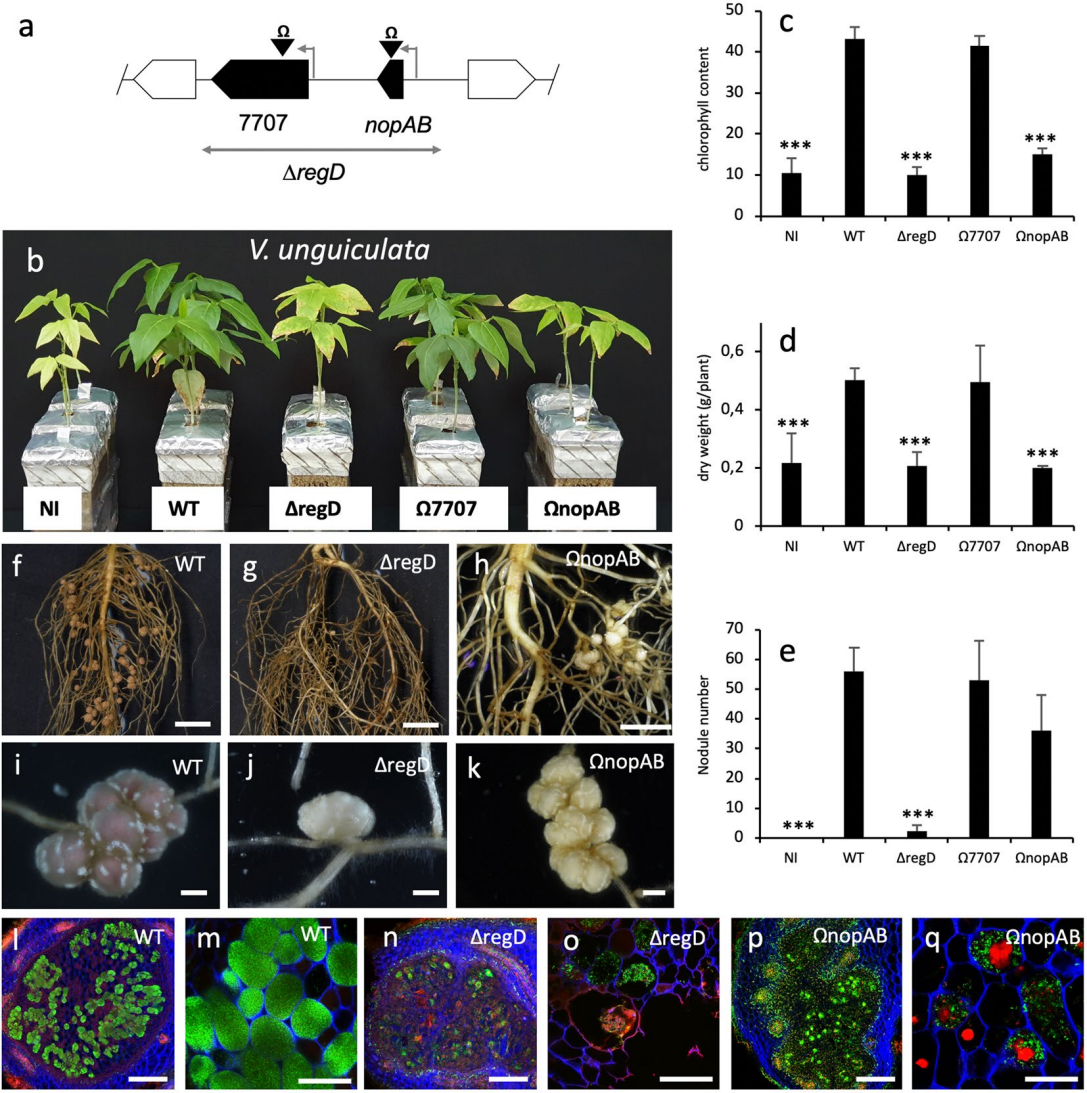
Symbiotic properties of the ORS3257 mutants in the different effectors of the region D in *V. unguiculata*. (**a**) Genetic organisation of the putative effectors and *tts* boxes identified in region D. The deleted region in the mutant is indicated by a double direction arrow. The insertion mutants are indicated by black arrowheads carrying the Ω sign. In black, putative effector genes; gray arrows, *tts* boxes. (**b**) Comparison of the growth of the plants (aerial part), non-inoculated (NI) or inoculated with ORS3257 wild-type strain (WT) or its derivative mutants at 21 days after inoculation. (**c**) Leaf chlorophyll content. (**d**) Plant dry weight; (**e**) Number of nodules per plant. The experiment was carried out in duplicate with five plants per condition. *P < 0.05, **P < 0.01, and ***P < 0.001, significant differences between WT ORS3257 and its derivative mutants using a nonparametric Kruskal–Wallis test. (**f**–**k**) View of the root and the nodules induced by strain ORS3257 and its derivative mutants. (Scale bars: f to h, 1 cm; i to k, 2 mm.) (**l**–**q**) Cytological analysis of the nodules induced by strain ORS3257 and its derivative mutants observed by confocal microscopy after staining with SYTO 9 (green: live bacteria), calcofluor (blue: plant cell wall), and propidium iodide (red: infected plant nuclei and dead bacteria or bacteria with compromised membranes). (Scale bars, l, n, p, 200 µm; m, o, q, 50 µm).

The region deleted in the 3257∆*regD* mutant corresponds to a small region (3.5 kb) containing two putative effector genes (*nopAB* and *Brad3257_7707*) (Fig. 4a). The analyses of the two available insertional mutants in these effectors showed that the mutation in *Brad3257_7707* did not lead to any particular phenotype whereas the symbiotic properties of the Ω*nopAB* mutant were strongly affected in the two *Vigna* species tested (Fig. 4b–k and Supplementary Fig. S3b–k). However, as observed for the ∆*regB* and Ω*nopT* mutants, the absence of the *nopAB* gene alone cannot explain the phenotype of the ∆*regD* mutant. Indeed, the ∆*regD* mutant produced fewer nodules than the Ω*nopAB* mutant (Fig. 4e and Supplementary Fig. S3e). Furthermore, the ∆*regD* nodules in both *Vigna* species displayed the same irregular infection as that observed in ∆*regB* nodules, while such a defect of infection appeared less marked in the Ω*nopAB* nodules, in particular in *V. mungo* (Fig. 4l–q and Supplementary Fig. S3l–q)*.* It is therefore possible that the more drastic phenotype of the ∆*regD* mutant was due to a cumulative effect of deleting *nopAB* and *Brad3257_7707* genes*.*

We also analysed the symbiotic phenotypes of two sub-mutants (Ω*nopP*1 and Ω*nopM1)* of region A that are specifically mutated in one of the two effectors identified in this region (Supplementary Fig. S4a). While the two mutants showed no particular phenotypes when tested on *V. unguiculata,* when tested on *V. mungo* both showed a significant deficiency in some of the symbiotic parameters measured (Supplementary Fig. S4b–g). This strongly suggests that the weak phenotype observed in the ∆*regA* mutant in *V. mungo* resulted from the combined absence of NopP1 and NopM1.

Taken together, these data indicate that both NopT and NopAB play a major positive role in the symbiotic interaction between ORS3257 and the two *Vigna* species *V. unguiculata* and *V. mungo*. But beyond these two T3Es, we hypothesise that other effectors such as NopP1, NopM1, Brad3257_7707 and very probably others that remain to be identified, also play a role in the global symbiotic performance of the strain, in particular in *V. mungo*.

### NopP2 blocks the symbiotic interaction between ORS3257 and *V. radiata* cv. SUT1

**Figure 5:**
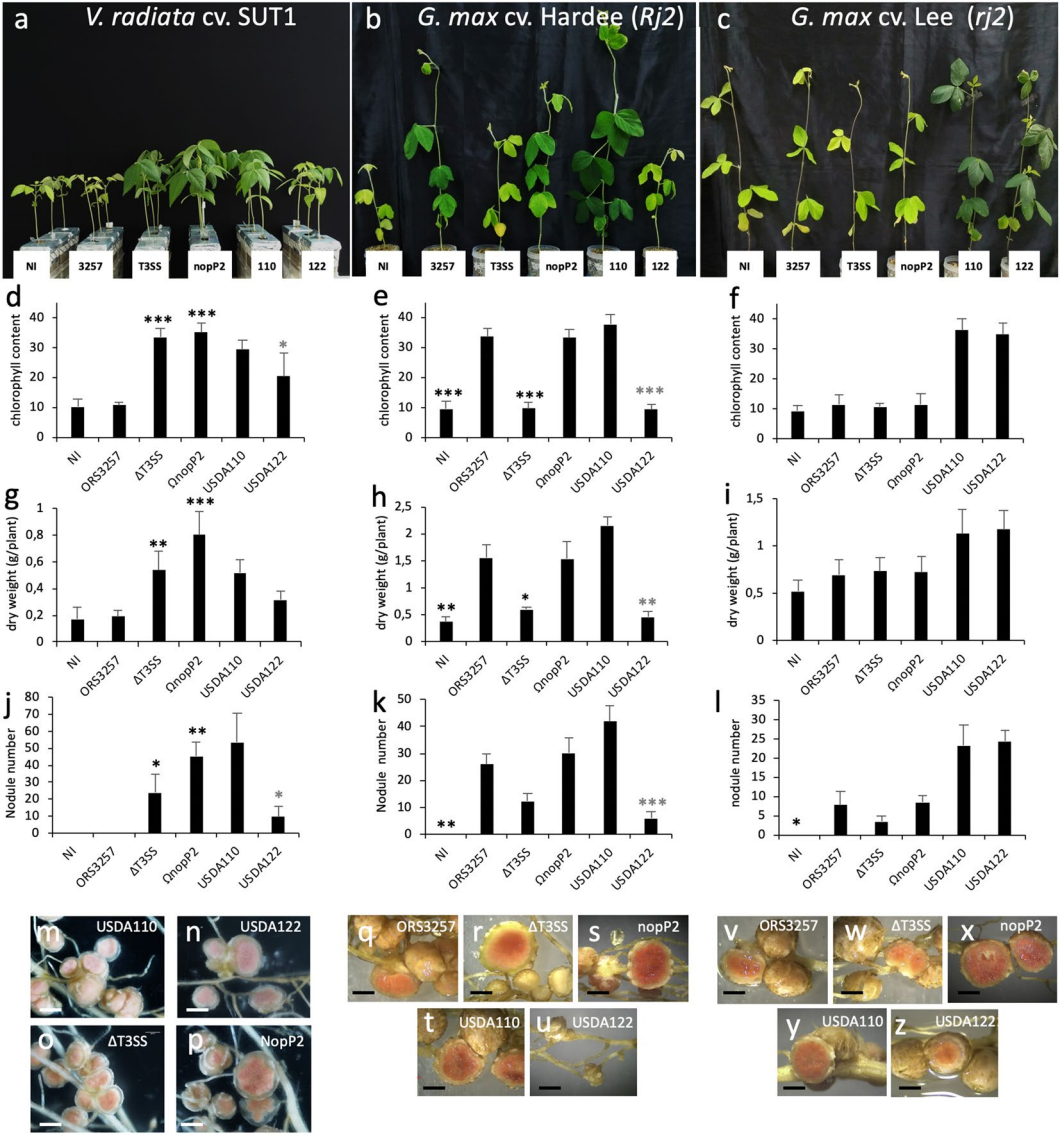
Symbiotic properties of various *Bradyrhizobium* strains in *V. radiata* cv. SUT1, *G. max* cv. Hardee (*Rj2*) and *G. max* cv. Lee (*rj2*). (**a**–**c**) Comparison of the growth of the plants (aerial part), non-inoculated (NI) or inoculated with ORS3257 wild-type strain (3257) or its derivative mutants (∆T3SS and ΩNopP2) or with USDA122 (122) or USDA110 (110) WT-strains at 21 days after inoculation. (**d–f**) Leaf chlorophyll content; (**g**–**i**) Plant dry weight; (**j–l**) Number of nodules per plant. The experiment was carried out in duplicate with five plants per condition. *P < 0.05, **P < 0.01, and ***P < 0.001. Black asterisks indicate significant differences between WT ORS3257 and other strains tested, using a nonparametric Kruskal–Wallis test, Gray asterisks indicate significant differences between WT USDA110 and WT USDA122. (**m**–**z**) View of the nodules. (Scale bars, 1 mm).

As done previously, we tested the 10 constructed mutants on *V. radiata* cv. SUT1 to identify the effector(s) which compromise the symbiosis of ORS3257 with this *Vigna* species. All the mutants tested displayed the same strict *nod* minus phenotype as observed in the WT strain except for the Ω*nopP2* mutant (Fig. 2g–i). Like the ∆*T3SS* mutant, the *nopP2* mutant resulted in functional pink nodules as indicated by improved plant growth and increased leaf chlorophyll content (Fig. 5a,d,g,j,o,p). These data clearly show that the effector NopP2 is responsible for the symbiotic incompatibility between ORS3257 and *V. radiatia* cv. SUT1.

Interestingly, NopP was also recently identified in *B. diazoefficiens* USDA122 as the causal effector that prevents the symbiotic interaction between this strain and *Rj2*-Soybeans^14^. Surprisingly, by swapping the *nopP* gene in USDA122 with the one of the compatible *B. diazoefficiens* USDA110 strain in which the corresponding protein differs in only four amino acid residues, it has been possible to restore a symbiotic interaction, indicating that the plant can reject or accept a compatible symbiont simply by monitoring some minor variations in NopP^14^. Interestingly, the ORS3257 strain contains two *nopP* homologues, *Brad3257_6978 (nopP1)* in region A, and *Brad3257_7831* (*nopP2*) elsewhere on the symbiotic island (Supplementary Fig. S1). The *∆regA* mutant was unable to form nodules, indicating that NopP1 is not involved in limiting this symbiosis (Fig. 2i), which we confirmed by also testing a specific insertional mutant (Ω*nopP1*) that also displayed a strict *nod* minus phenotype (Fig. 2i). Sequence alignment between the different NopP sequences showed that NopP2 shared higher identity with the NopP from USDA122 and USDA110 (71.9% of identity with each) than with NopP1 (40.6% and 39.9% of identity with the NopP of USDA122 and USDA110, respectively) (Supplementary Fig. S5). However, two out of the three AA of USDA122 NopP shown to be required for *Rj2*-soybean incompatibility are not conserved in NopP2 (Supplementary Fig. S5).

To investigate whether the mechanisms governing the incompatibility between ORS3257 and *V. radiata* cv. SUT1 are the same as those described between USD122 and *Rj2* soybeans, we first analysed the symbiotic properties of USDA110 and USDA122 strains in *V. radiata* cv. SUT1. USDA110 was clearly seen to be more efficient than USDA122 in this cultivar, i.e. more nodules were formed, and plant growth and chlorophyll content were higher (Fig. 5a,d,g,j). But at the same time, this difference was not as black and white as the difference observed between ORS3257 and Ω*nopP2.* Indeed, the nodules elicited by USDA110 and USDA122 look similar, both were pink and we even observed a small improvement in growth and chlorophyll content in the plants inoculated with USDA122 (Fig. 5d,g,m,n).

The ORS3257 WT strain and its ∆*T3SS* and Ω*nopP2* mutants were also tested on *Rj2* and *rj2* soybeans, and USDA110 and USDA122 strains were also included as controls. We confirmed the inability of USDA122 to efficiently nodulate *Rj2*-soybean ‘Hardee’, only a few small pseudo-nodules were observed and no improvement in plant fitness was measured (Fig. 5b,e,h,k,t,u), whereas when tested on *rj2*-soybean ‘Lee’, both strains displayed similar symbiotic efficiency (Fig. 5c,f,i,l,y,z). Unexpectedly, the ORS3257 WT strain and its Ω*nopP2* mutant were found to be symbiotically efficient in *Rj2*-soybean ‘Hardee’ while the ∆*T3SS* mutant induced fewer nodules that had no apparent benefit for plant growth and chlorophyll content (Fig. 5b,e,h,k,q,r,s). These data indicate that NopP2 protein of ORS3257 was not recognized as an incompatible effector by *Rj2*-soybean ‘Hardee’ and that by secreting other T3Es, T3SS played an important positive role in this symbiotic interaction. T3SS was also observed to play a weak positive role in the nodulation of *rj2*-soybean ‘Lee’, as ORS3257 and the Ω*nopP2* mutant resulted in more nodules than the ∆*T3SS* mutant (Fig. 5l). Nevertheless, in the latter case, the nodules elicited by WT ORS3257 and Ω*nopP2* were apparently not functional since no improvement was observed in plant growth or chlorophyll content despite the fact that the nodules displayed a pink colour (Fig. 5c,f,i,v,w,x).

Taken together, these data clearly show that NopP2 of ORS3257 blocks nodulation with *V. radiata* cv. SUT1 but it is still not clear whether this symbiotic incompatibility is induced via a R-protein homologous to Rj2 identified in soybeans.

## Discussion

A recent phylogenomic analysis conducted on a large number of *Bradyrhizobium* genomes showed that a T3SS is almost systematically found in all the strains nodulating and containing *nod* genes, suggesting that T3SS is an overriding factor in determining the symbiotic properties of the rhizobia that belong to this genus^10^. Our study conducted on *B. vignae* ORS3257 strain supports this hypothesis as we observed drastic impacts of the mutation of the T3SS machinery on the symbiosis of the bacteria on the three *Vigna* species tested. Interestingly, and as already reported for several *Bradyrhizobium* or rhizobium strains belonging to other genera^11^, depending on the host plant, T3SS can either support symbiotic efficiency, or quite the reverse, completely block the symbiosis.

By using a collection of mutants affected in the effector genes identified in this strain, we showed that a combination of different numbers of effectors is required to support the efficiency of the symbiosis of the ORS3257 strain in *V. unguiculata* and *V. mungo*, while only one effector NopP2 is sufficient to block the symbiosis with *V. radiata* cv. SUT1. Interestingly, NopT and NopAB, which were found to play an important role in the symbiosis of ORS3257 with *V. unguiculata* and *V. mungo*, as well as NopP1 and NopM1, which were found to play a positive role in *V. mungo* although to a lesser extent*,* were previously shown to be required for the establishment of the NF-independent symbiosis with *Aeschynomene indica*^17^. Conversely, ErnA, which was the key effector triggering nodulation on *A. indica*, did not play a significant role in these two *Vigna* species. The NFs signal synthetized by ORS3257 strain, which we showed to be absolutely indispensable for the symbiosis in the two latter species, most probably renders the role of ErnA in nodule organogenesis superfluous. However, from these observations, we are still not able to rule that ErnA is dispensable in all the symbioses involving NFs, given the very high occurrence of this gene in *Bradyrhizobium* strains containing *nod* genes and its high level of conservation^10,17^. It is possible that ErnA strengthens or replaces the NF signal in certain culture conditions or during interactions with particular hosts.

The NopT and NopAB mutants induced fewer nodules and some disorders in intracellular infection were observed, particularly in *V. unguiculata*. In *A. indica*, it was previously reported that the nodules formed by these two mutants were completely devoid of infected central tissue, thereby pointing to an important role for these effectors in bacterial infection^17^. The importance of NopT in the modulation of the symbiotic interaction has also already been observed in *Enisifer fredii* NGR234. Interestingly, in this strain, NopT has been shown to act as a ‘two-edge sword’ which affected nodulation either positively in *Phaseolus vulgaris* or negatively in *Crotalaria juncea*^22^. The NopT effectors identified in rhizobia belong to the YopT-AvrPphB cysteine protease family initially characterised in pathogenic bacteria. Several functional characterisation of the NopT from diverse rhizobia (*E. fredii* NGR234, *Mesorhizobium amphore* and *B. diazoefficiens*) show that this T3E displays autoproteolytic activity, targets the plasma membrane of the host cell most probably through its lipid acylation after its translocation, and elicits HR-like cell death when expressed in tobacco^22–24^. Very recently, two possible targets of the NopT of *M. amphore* CCNWGS0123 were identified in *Robinia pseudoacacia*, an ATP-citrate synthase and a hypersensitive induced response protein (HIRP)^24^. However, how the interaction of NopT with these targets can modulate the symbiotic process remains to be clarified.

Knowledge of the NopAB effector is still extremely limited. This effector family was originally identified in several *B. diazoefficiens* strains through an in silico search of genome sequences of genes associated with the regulatory *tts*-box motif^25^. Sequence analysis of NopAB did not enable prediction of any functional domains and no homolog has been found outside of the bradyrhizobia, suggesting that NopAB is a specific effector of unknown function that emerged in this genus. Interestingly, an analysis of its distribution in the *Bradyrhizobium* genus showed that NopAB is very well conserved since more than 50% of the strains containing a T3SS have a NopAB homolog (47 out of 92 strains)^10^. However, to our knowledge, no other study has reported a role for this effector in other *Bradyrhizobium* strains. Considering the important role played by NopAB in ORS3257 during the interaction with several legume species, it would be interesting to further explore its function and examine its symbiotic role in other strains. To a lesser extent, we also observed that NopM1 and NopP1 play a significant role during the symbiotic interaction of ORS3257 and *V. mungo*. These two effectors were also shown to play a positive role during the interaction between *E. fredii* NGR234 and several legumes species^26,27^, while in *B. elkanii* USDA61 it has been recently reported that NopM induces a nodule early senescence-like response in *Lotus burttii* and *L. japonicus* MG-20^28^. The mode of action of NopP remains unclear. It has been shown that the NopP of NGR234 can be phosphorylated in vitro by plant kinases but it is still not known whether this phosphorylation suppresses plant defence responses by interfering with MAP kinase signalling pathways as has been shown for NopL, another effector in NGR234, which also plays a positive symbiotic role in certain host plants^29,30^. The NopM effector of NGR234 has recently been shown also to be phosphorylated by plant kinase^31^. Interestingly, this effector displays moreover an E3 ubiquitin ligase activity^31,32^. It has been hypothesised that NopM promotes nodulation by ubiquitination of specific host proteins to target them for proteasome-dependent proteolysis and/or by interfering with the MAP signalling pathways, like NopL^31^. Similarly, the NopM1 and NopP1 identified in ORS3257 could interfere via phosphorylation and ubiquitination and act synergistically to suppress plant defences and subsequently favours nodulation of *V. mungo*. It is important to note that ORS3257 possesses another homolog of NopP (NopP2) and NopM (NopM2). The Ω*nopP2* mutant was found to be slightly affected during the symbiosis with *V. mungo* (Fig. 2d,e), in combination with NopP1 and NopM1, NopP2 could therefore also be involved in the modulation of plant immunity. In the case of NopM2, at this stage, in the absence of a specific mutant in this gene, it is difficult to argue whether or not this effector has a cumulative effect, even if the *∆regC* mutant, which includes the deletion of *nopM2*, displayed no specific phenotype (see discussion below).

Several observations lead us to assume that during the screening of this collection of mutants, we missed certain effectors perhaps involved in the modulation of the symbiotic properties of ORS3257. One observation is the fact that the phenotype of the ∆*T3SS* mutant, in which the secretory machinery is completely abolished, was not as drastic as in other mutants in some deleted regions (∆*regB* and ∆*regD*) or in specific effectors (Ω*nopT* and Ω*nopAB*). In the ∆*T3SS* mutant, all the possible effectors that could have either a positive or a negative effect were eliminated. We therefore hypothesise that the less drastic phenotype observed results from the suppression of effectors playing a negative role. However, none of the mutants tested showed a symbiotic gain. It cannot be ruled out that the mutagenesis approach we used, which consisted in deleting regions containing several *T3E*s, has certain limits if effectors in this same region have antagonistic effects on the symbiosis. In particular, this could be the case of region C that contains nine T3Es and for which the corresponding mutant ∆*regC* displayed no particular phenotype. In the same line of thought, we observed that the ∆*regB* and ∆*regD* mutants had a more drastic phenotype than the Ω*nopT* and Ω*nopAB* mutants, suggesting that the absence of other effectors or some genetic determinants present in these regions also contribute to the phenotype of the two deleted mutants. But at the same time, the mutation of the other candidate effectors (*Brad3257_7707*, *nopAO)*, or the deletion of the regions encompassing the two *tts* boxes (*∆regB1* and *∆regB2*) did not lead to a specific phenotype. It is possible that some effectors or determinants in these two regions make an incremental contribution with NopT and NopAB in the global symbiotic performance of the strain but that their specific effect remains limited.

Surprisingly, the effectors identified as playing an important role during the symbiotic interaction between ORS3257 and *V. mungo* are not the same than the ones recently identified in *B. elkanii* USDA61 interacting with the same species^33^. In particular, T3SS was shown to be essential in USDA61 during this interaction and the T3E shown to play a major positive role is NopL, while the Ω*nopL* mutant of ORS3257 displayed no specific phenotype in this plant species. Similarly, NopAB, which is important in ORS3257, is absent in USDA61. For NopT, the question remains whether this effector plays a similar role in USDA61 for which a homolog has been found but, to our knowledge, no mutant has yet been tested in *V. mungo*.

On the other hand, USDA61 was also shown to be incompatible on *V. radiata* cv. KPS1, but the causal effector that blocks this interaction is not NopP but another effector named InnB for (Incompatible nodulation B)^34,35^. It is to note that USDA61 possess two NopP homologs, one of which is very similar to NopP2 of ORS3257 (Supplementary Fig. S5). We cannot exclude the possibility that these NopP homologs have a negative effect during this interaction but if it exists, the effect is very likely limited given that *innB* mutant is highly efficient^35^. The observed differences between the two strains could be explained by the fact that the plant cultivar used in the two studies was different, but also by the fact that each strain has evolved and adapted its effectome according to the different plant partners encountered during its history.

It has been proposed that the molecular mechanisms mediated by recognition of NopP by Rj2 protein leading to the incompatibility between some *Bradyrhizobium* strains and some soybean cultivars are conserved in other legumes^14^. Two arguments were put forward to support this hypothesis: (i) the identification of Rj2 orthologs in various legume genomes including in *V. radiata* and, (ii) the fact that a USDA122 mutant in which the *nopP* gene was swapped by the one of USDA110 was found to be more efficient in *V. radiata* cv KPS1 than the WT strain USDA122. The observation that NopP2 in ORS3257 blocks the interaction with another *V. radiata* ecotype is consistent with this view. Nevertheless, we observed that NopP2 of ORS3257 did not restrict the compatibility with the Rj2 soybean ‘Hardee’. It is possible that the Rj2 orthologs identified in *V. radiata* and soybeans evolved differently and recognize different specific NopP variations in order to monitor the compatibility or incompatibility of their symbionts. It would be interesting to investigate if ORS3257 is able to nodulate other *V. radiata* varieties and compare the Rj2 sequences between compatible and incompatible ecotypes.

While inoculation of soybean fields is a common practice to increase grain yields across the world, inoculation of mung bean, cowpea or black mung bean fields remains limited, especially in Africa^36^. Beside the low cost of chemical fertilizers which represents a real impediment to the development of sustainable agriculture, one limitation in the use of inoculant is the identification of effective strain that are more competitive than the endogenous microflora and can be used for a broader range of crops. Identifying the genetic determinants in Rhizobium which promote or compromise the symbiosis is important for the design or selection of appropriate inoculum strains.

## Methods

### Bacterial strains and culture conditions

Supplementary Table S1 lists the bacterial mutants of the ORS3257 strain used in this study. *Bradyrhizobium* sp. strains (ORS3257, USDA110 and USDA122) and the derivative mutants of ORS3257 were grown in yeast mannitol (YM) medium^37^ at 34 °C on a rotary shaker at 150 rpm. When required, the media were supplemented with appropriate antibiotics at the following concentrations: 20 µg/mL Nalidixic acid, 50 µg/mL kanamycin (Km), and 20 µg/mL cefotaxime (Cefo). The cefotaxime resistance of the mutants results to the use of the cefotaxime resistant gene originating from *E. coli* strain 25,104 as selective marker^16^.

### Construction of a nodABC mutant of ORS3257

For the construction of the 3257∆*nodABC* mutant, the upstream and downstream flanking regions (around 750 bp) of the *nodABC* genes were amplified using the primers GGGGGGGATCCAATAGCAAACATCAGTTTGGAAAAG (Up.NodA.3257.f), GAGCCAATCAAAGCTTGCCGGGTTTCCTTGCGCCAGTGGAAT (Up.NodA.3257.r), GAAACCCGGCAAGCTTTGATTGGCTCATGATTCCGGACGCAA (Dw.NodC.3257.f), and CGTTGCTCTAGATTTCTTGCTCGATGCAAAAGACAAG (Dw.NodC.3257.r). The PCR fragments were merged by overlap extension PCR and cloned into pNPTS129 at the BamHI/XbaI sites. Subsequently, a cefotaxime resistance cartridge was cloned between the upstream and downstream fragments in the HindIII site. The resulting plasmid was then transferred by conjugation into the ORS3257 strain and the deleted mutant obtained by double crossing over was selected as described previously^38^.

### Plant nodulation and symbiosis analysis

The plants used in this study are listed in Supplementary Table S2. The seeds of the different legume species tested were surface sterilised with a 3% (v/v) calcium hypochlorite solution for 3 min, washed with sterilised water and then soaked in 70% (v/v) ethanol for 3 additional min. Finally, the seeds were washed in sterilised water five times and left to germinate on 0.8% water agar plates at 34 °C overnight. The germinated seeds were transplanted into Leonard jars filled with sterilised vermiculite^39^ and watered with BNM medium^40^. The plantlets were grown under the following controlled environmental conditions: 28 °C with a 16-h light and 8-h dark regime at light intensities of 300 µmol/m^2^S and 70% humidity. Five days after planting, each seedling was inoculated with 1 mL of a 5-day old inoculum after washing and adjusting the optical density at 600 nm to 1 (approximately 10^9^ cells/mL). Twenty-one days after inoculation (dpi), the leaf chlorophyll content was monitored using a Minolta SPAD-502 chlorophyll meter^41^, the number of nodules was counted, and the dry weights of plants were determined after drying at 50 °C for 96 h. The experiment was carried out in duplicate using five plants per condition.

### Cytological analysis of nodules

Fresh nodules were observed under an optical Macroscope (Nikon AZ100, Champigny-sur-Marne, France). Sections (40–50 µm thick) of fresh nodules were prepared using a VT1200S vibratome (Leica Nanterre, France). Nodule sections were incubated for 15 min in live/dead staining solution (5 μM SYTO 9 and 30 μM propidium iodide in 50 mM Tris pH 7.0 buffer; Live/Dead BacLight, Invitrogen, Carlsbad, CA, USA). The sections were then removed and incubated for an additional 15 min in 10 mM phosphate saline buffer containing calcofluor white M2R (Sigma, Munich, Germany) at a final concentration of 0.01% (wt/vol) to stain the plant cell wall^42^. Analyses were carried out using a confocal laser-scanning microscope (Carl Zeiss LSM 700, Jena, Germany). Calcofluor was excited at 405 nm with emission signal collection at 405–470 nm. For SYTO 9 and propidium iodide, an excitation wavelength of 488 and 555 nm was used to collect emission signals at 490–522 nm and 555–700 nm, respectively. Images were obtained using the ZEN 2008 software (Zeiss, Oberkochen, Germany).

## Supplementary Information

Supplementary Information.
