# Publisher Correction: Identification of type III effectors modulating the symbiotic properties of *Bradyrhizobium vignae* strain ORS3257 with various *Vigna* species

**DOI:** 10.1038/s41598-021-91376-z

**Published:** 2021-06-11

**Authors:** Pongpan Songwattana, Clémence Chaintreuil, Jenjira Wongdee, Albin Teulet, Mamadou Mbaye, Pongdet Piromyou, Djamel Gully, Joel Fardoux, Alexandre Mahougnon Aurel Zoumman, Alicia Camuel, Panlada Tittabutr, Neung Teaumroong, Eric Giraud

**Affiliations:** 1 School of Biotechnology, Institute of Agricultural Technology, Suranaree University of Technology, Nakhon Ratchasima, 30000 Thailand; 2 IRD, Laboratoire des Symbioses Tropicales et Méditerranéennes, UMR 113, IRD/CIRAD/INRAE/Université de Montpellier/SupAgro, Campus de Baillarguet, TA‑A82/J, 34398 Montpellier Cedex 5, France; 3 IRD, Laboratoire Commun de Microbiologie, UR040, ISRA, UCAD, Centre de Recherche de Bel Air, Dakar, Senegal; 4 Département de Biologie Végétale, University Cheikh Anta Diop, Dakar, Senegal

Correction to: *Scientific Reports* https://doi.org/10.1038/s41598-021-84205-w, published online 01 March 2021

The original PDF version of this Article contained an error where part of the sentence “Considering that the T3SS has been shown to bypass the requirement of NF signalling during the interactions of some *Bradyrhizobium* strains including ORS3257 with some tropical legumes^15,16^, we constructed a mutant in which the *nodABC* genes were deleted and analysed its symbiotic properties.” was incorrectly placed next to Figure 1.

This error has now been corrected in the PDF version of the Article; the HTML version was correct from the time of publication.

