# Supplementary material for: Identification of type III effectors modulating the symbiotic properties of Bradyrhizobium vignae strain ORS3257 with various Vigna species
Source: Sci Rep. 2021 Mar 1;11:4874. doi: 10.1038/s41598-021-84205-w (PMC7921652; doi:10.1038/s41598-021-84205-w)
Supplement: Supplementary file 1 — Supplementary Information. [file 41598_2021_84205_MOESM1_ESM.pdf]

Supplementary Information

Identification of type III effectors modulating the symbiotic properties of *Bradyrhizobium vignae* strain ORS3257 with various *Vigna* species

Authors: Pongpan Songwattana[1]‡, Clémence Chaintreuil[2,3]‡, Jenjira Wongdee[1], Albin Teulet[2], Mamadou Mbaye[2], Pongdet Piromyou[1], Djamel Gully[2], Joel Fardoux[2], Alexandre Mahougnon Aurel Zoumman[3,4], Alicia Camuel[2], Panlada Tittabutr[1], Neung Teaumroong[1*], Eric Giraud[2*].

*Correspondence: Neung Teaumroong, neung@sut.ac.th; Eric Giraud, eric.giraud@ird.fr

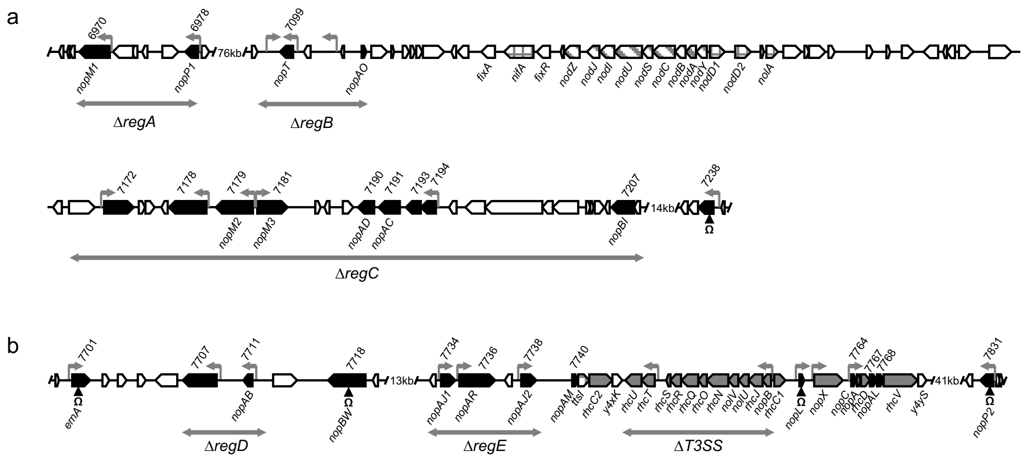

Figure S1: Genetic organisation of the putative type III effectors of *Bradyrhizobium vignae* ORS3257 strain.
The type III secretion related genes are located in two distinct genetic loci, one from each side of the symbiotic island: from 6,481,200 bp to 6,671,200 bp (A) and from 7,012,800 bp to 7,120,700 bp (B). Deleted regions in the mutants are indicated by gray double direction arrows. Insertion mutants are indicated by black arrowheads carrying the Ω sign. Black and gray ORFs represent genes encoding putative effectors and components of the T3SS apparatus, respectively. Hatched ORFs: *nod* genes; Gray arrows: *tts* boxes.

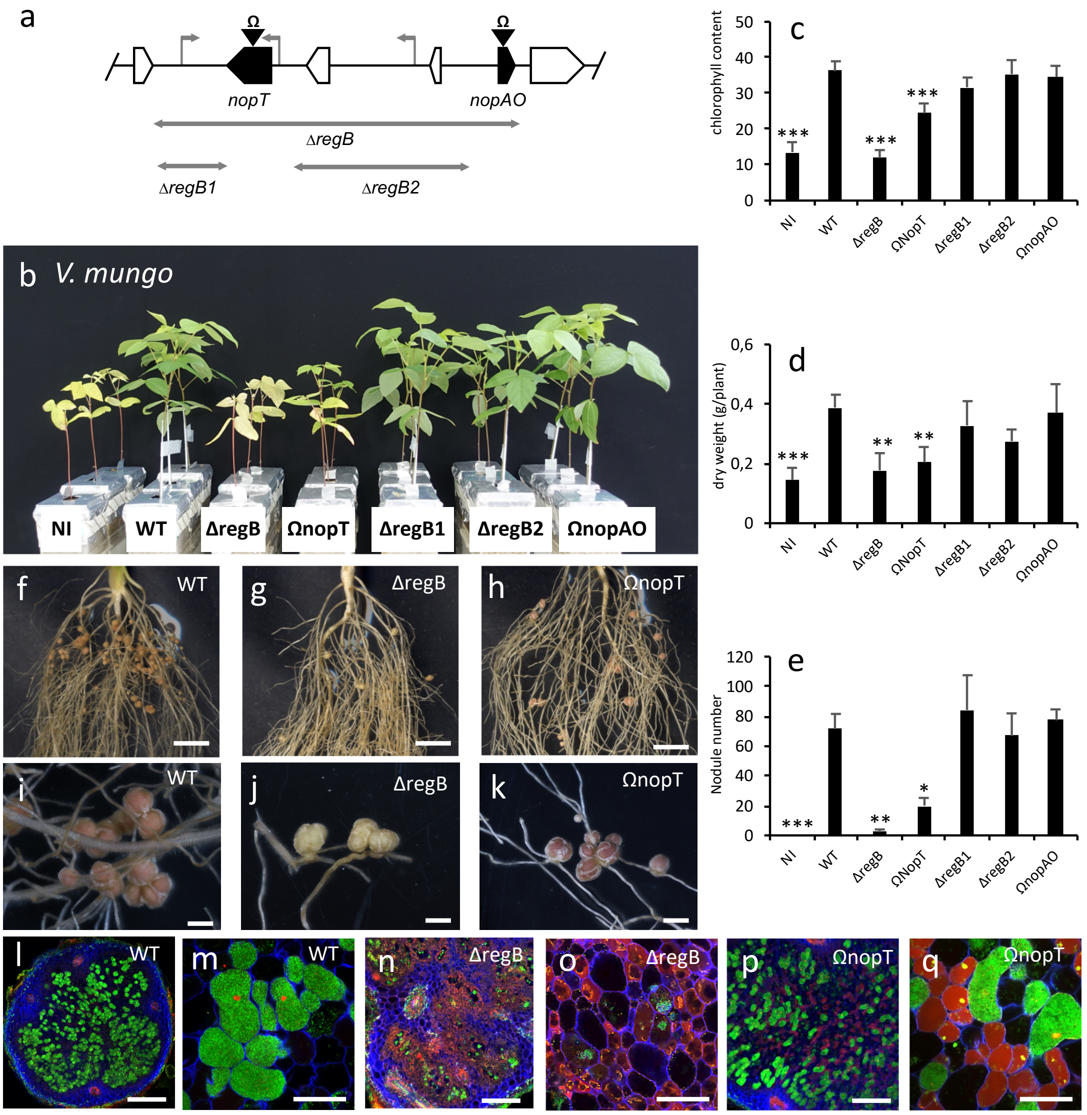

Figure S2: Symbiotic properties on *V. mungo* of the ORS3257 mutants in the different effectors and the regions encompassing the *tts* boxes identified in region B.

a. Genetic organisation of the putative effectors and *tts* boxes identified in region B. The deleted region in the mutants are indicated by double direction arrows. The insertion mutants are indicated by black arrowheads carrying the Ω sign. In black, putative effector genes; gray arrows, *tts* boxes. **b.** Comparison of the growth of the plants (aerial part), non-inoculated (NI) or inoculated with ORS3257 wild-type strain (WT) or its derivative mutants at 21 days after inoculation. **c.** Leaf chlorophyll content (SPAD unit). **d.** Plant dry weight; **e.** Nodule number per plant. The experiment was carried out in duplicate with five plants per condition; *P < 0.05, **P < 0.01, and ***P < 0.001, significant differences between WT ORS3257 and its derivative mutants using a nonparametric Kruskal–Wallis test. **f to k**. View of the root and the nodules induced by strain ORS3257 and its derivative mutants. (Scale bars: f to h, 1 cm; i to k, 2 mm). **l to q.** Cytological analysis of the nodules induced by strain ORS3257 and its derivative mutants observed by confocal microscopy after staining with SYTO 9 (green; live bacteria), calcofluor (blue; plant cell wall), and propidium iodide (red; infected plant nuclei and dead bacteria or bacteria with compromised membranes). (Scale bars, l, n, p, 200 µm; m, o, q, 50 µm).

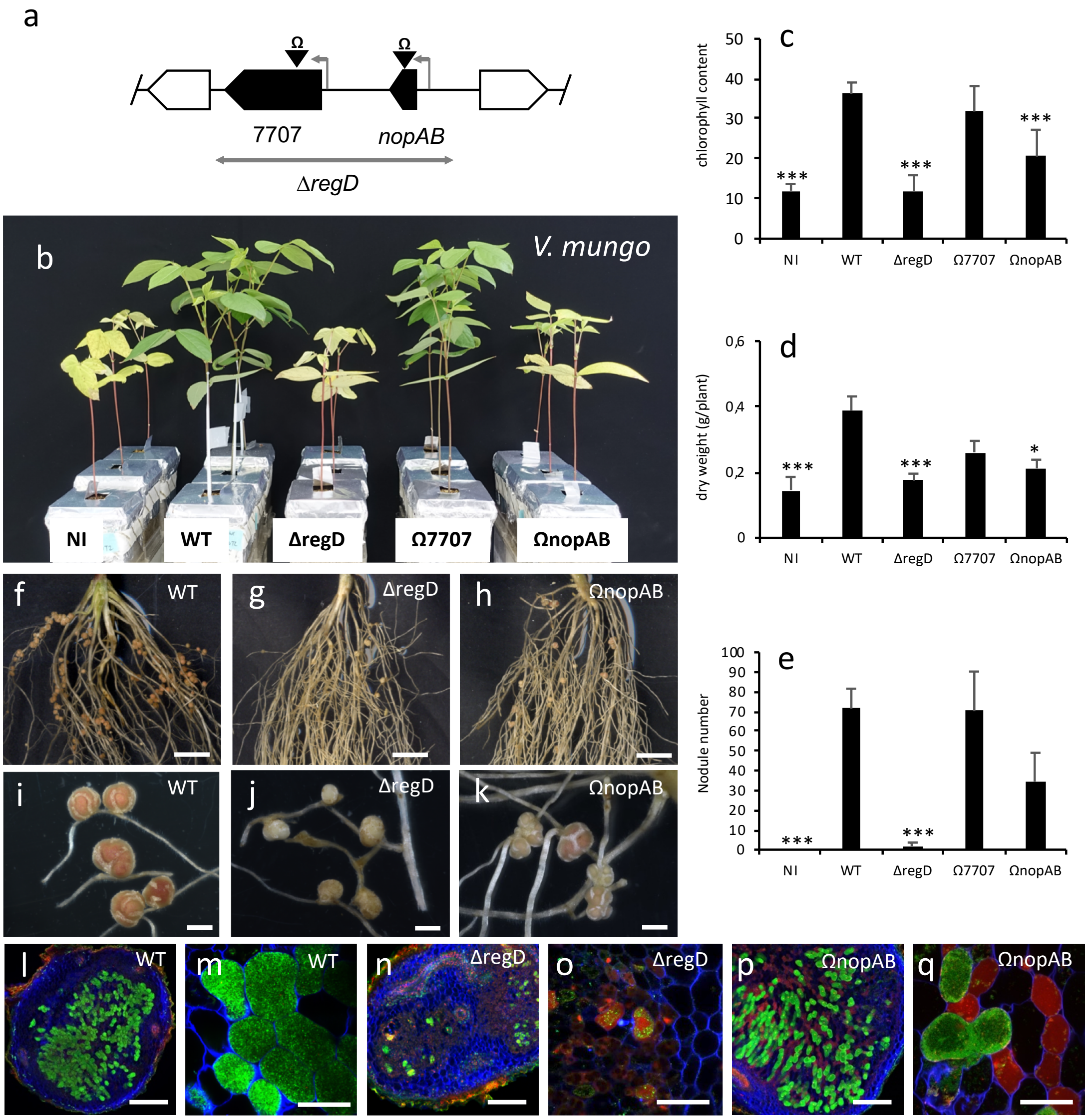

Figure S3: Symbiotic properties of the ORS3257 mutants in the different effectors of the region D in *V. mungo*.

a. Genetic organisation of the putative effectors and *tts* boxes identified in region D. The deleted region in the mutant is indicated by a double direction arrow. The insertion mutants are indicated by black arrowheads carrying the Ω sign. In black, putative effector genes; gray arrows: *tts* boxes. **b.** Comparison of the growth of the plants (aerial part), non-inoculated (NI) or inoculated with ORS3257 wild-type strain (WT) or its derivative mutants at 21 days after inoculation. **c.** Leaf chlorophyll content. **d.** Plant dry weight; **e.** Number of nodules per plant. The experiment was carried out in duplicate with five plants per condition*P < 0.05, **P < 0.01, and ***P < 0.001, significant differences between WT ORS3257 and its derivative mutants using a nonparametric Kruskal–Wallis test. **f to k**. View of the root and the nodules induced by strain ORS3257 and its derivative mutants. (Scale bars: f to h, 1 cm; i to k, 2 mm.) **l to q.** Cytological analysis of the nodules induced by strain ORS3257 and its derivative mutants observed by confocal microscopy after staining with SYTO 9 (green: live bacteria), calcofluor (blue: plant cell wall), and propidium iodide (red: infected plant nuclei and dead bacteria or bacteria with compromised membranes). (Scale bars, l, n, p, 200 µm; m, o, q, 50 µm).

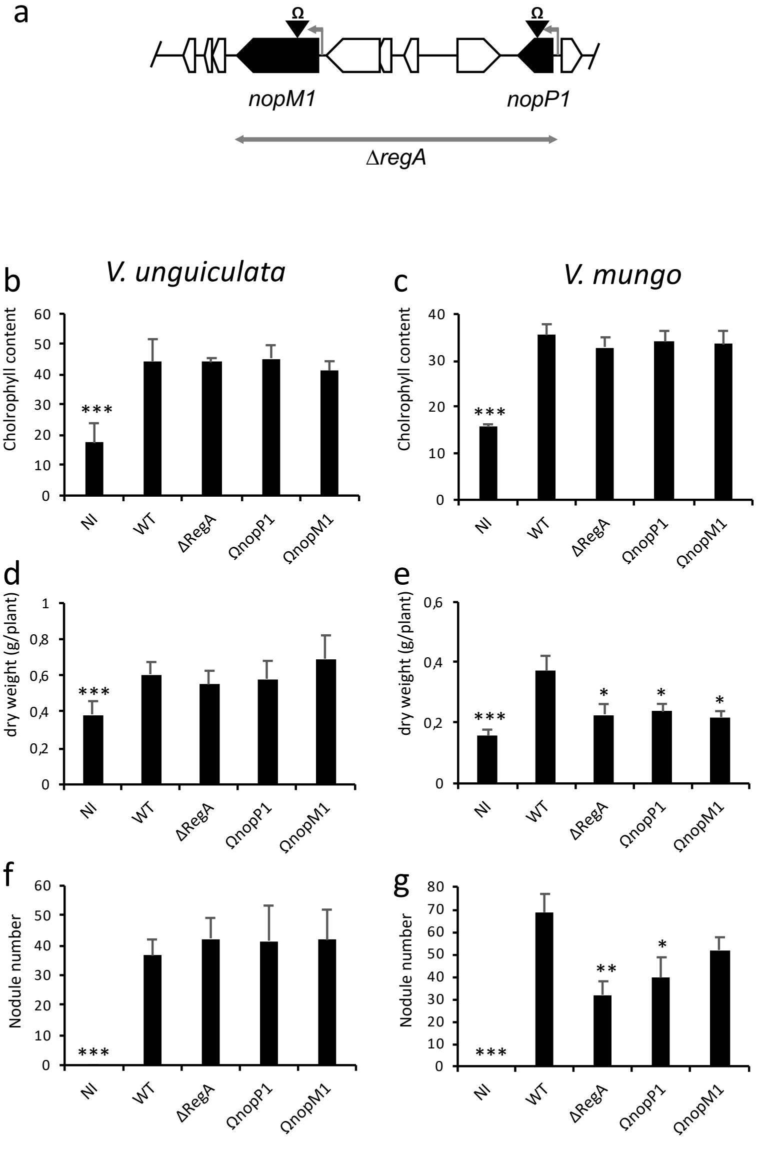

Figure S4: Symbiotic properties of the ORS3257 mutants in the different effectors of the region A in *V. unguiculata* and *V. mungo*.
A. Genetic organisation of the putative effectors and *tts* boxes identified in region A. The deleted region is indicated by a double direction arrow. The insertion mutants are indicated by black arrowheads carrying the Ω sign. In black, putative effector genes; gray arrows: *tts* boxes. **B, C.** Leaf chlorophyll content of the plants non-inoculated (NI) or inoculated with ORS3257 wild-type strain (WT) or its derivative mutants at 21 days after inoculation. **D, E.** Plant dry weight **F, G.** Number of nodules per plant. The experiment was carried out in duplicate with five plants per condition*P < 0.05, **P < 0.01, and ***P < 0.001, significant differences between WT ORS3257 and the derivative mutants using a nonparametric Kruskal–Wallis test.

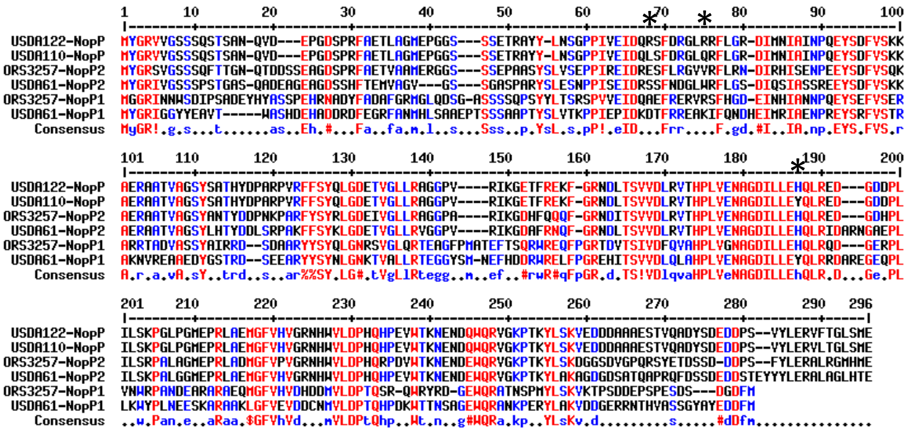

Figure S5: Comparison of NopP effectors between *B. vignae* ORS3257, *B. diazoefficiens* USDA 110 and USDA 122 and *B. elkanii* USDA 61.

The NopP sequences were compared using the multalin programme (http://multalin.toulouse.inra.fr/multalin/cgi-bin/multalin.pl). Asterisks indicate the three amino acid residues (R60; R67 and H173) in USDA 122 NopP required for Rj2-Mediated incompatibility in soybean (Sugawara et al. 2018). Accession number of the NopP sequences: USDA 122-NopP, APO50372; USDA 110-NopP, AAG60738; ORS3257-NopP1, SPP97835; ORS3257-NopP2, SPP98457; USDA 61-NopP1, BBC02248; USDA 61-NopP2, BBC02610.

Table S1: Bacterial strains used in this study

Strains [a]	Characteristics [b]	Reference
ORS3257	Wild-type strain *B. vignae*	
USDA110	Wild-type strain *B. diazoefficiens*	
USDA122	Wild-type strain *B. diazoefficiens*	
Δ*regA*	ORS3257 derivative mutant with the region A deleted via double crossing-over, Cf^r	17
Δ*regB*	ORS3257 derivative mutant with the region B deleted via double crossing-over, Cf^r	17
Δ*regB1*	ORS3257 derivative mutant with the region B1 deleted via double crossing-over, Cf^r	17
Δ*regB2*	ORS3257 derivative mutant with the region B2 deleted via double crossing-over, Cf^r	17
Δ*regC*	ORS3257 derivative mutant with the region C deleted via double crossing-over, Cf^r	17
Δ*regD*	ORS3257 derivative mutant with the region D deleted via double crossing-over, Cf^r	17
Δ*regE*	ORS3257 derivative mutant with the region E deleted via double crossing-over, Cf^r	17
Δ*T3SS*	ORS3257 derivative mutant of main genes encoding components of the T3SS apparatus deleted via double crossing-over, Cf^r	17
Δ*nodABC*	ORS3257 derivative mutant with the canonical *nodABC* genes deleted via double crossing-over, Cf^r	This study
Ω*nopM1*	ORS3257 derivative mutant with insertion of the plasmid pVO155-npt2-GFP-npt2-Cefo in the *nopM1* gene via simple crossing-over, Cf^r, Km^r	17
Ω*nopP1*	ORS3257 derivative mutant with insertion of the plasmid pVO155-npt2-GFP-npt2-Cefo in the *nopP1* gene via simple crossing-over, Cf^r, Km^r	17
Ω*nopT*	ORS3257 derivative mutant with insertion of the plasmid pVO155-npt2-GFP-npt2-Cefo in the *nopT* gene via simple crossing-over, Cf^r, Km^r	17
Ω*nopAO*	ORS3257 derivative mutant with insertion of the plasmid pVO155-npt2-GFP-npt2-Cefo in the *nopAO* gene via simple crossing-over, Cf^r, Km^r	17
Ω*7238*	ORS3257 derivative mutant with insertion of the plasmid pVO155-npt2-GFP-npt2-Cefo in the *Brad3257_7238* gene via simple crossing-over, Cf^r, Km^r	17
Ω*ernA*	ORS3257 derivative mutant with insertion of the plasmid pVO155-npt2-GFP-npt2-Cefo in the *ernA* gene via simple crossing-over, Cf^r, Km^r	17
Ω*7707*	ORS3257 derivative mutant with insertion of the plasmid pVO155-npt2-GFP-npt2-Cefo in the *Brad3257_7707* gene via simple crossing-over, Cf^r, Km^r	17
Ω*nopAB*	ORS3257 derivative mutant with insertion of the plasmid pVO155-npt2-GFP-npt2-Cefo in the *nopAB* gene via simple crossing-over, Cf^r, Km^r	17
Ω*nopBW*	ORS3257 derivative mutant with insertion of the plasmid pVO155-npt2-GFP-npt2-Cefo in the *nopBW* gene via simple crossing-over, Cf^r, Km^r	17
Ω*nopL*	ORS3257 derivative mutant with insertion of the plasmid pVO155-npt2-GFP-npt2-Cefo in the *nopL* gene via simple crossing-over, Cf^r, Km^r	17
Ω*nopP2*	ORS3257 derivative mutant with insertion of the plasmid pVO155-npt2-GFP-npt2-Cefo in the *nopP2* gene via simple crossing-over, Cf^r, Km^r	17

[a] Δ, deletion mutants; Ω, insertional mutants. [b] Cf^r, cefotaxime resistant; Km^r, kanamycin resistant.

Table S2: Plant species used in this study

Species	Cultivar	Origin
Vigna unguiculata (L.) Walp	Mélakh	Senegal
Vigna mungo (L.) Hepper	Uthong 2	Thailand
Vigna radiata (L.) Wilczek	SUT1	Thailand
Glycine max (L.) Merr-*RJ2*	Hardee	USA
Glycine max (L.) Merr-*rj2*	Lee	USA
